# Complete genome sequences of *Klebsiella pneumoniae* bacteriophages YMR1 and YMR2 isolated from sewage

**DOI:** 10.1128/mra.00544-24

**Published:** 2024-07-31

**Authors:** Miri Kwon, Yonghyun Lee, Minsik Kim

**Affiliations:** 1 Department of Food and Nutrition, Yonsei University, Seoul, South Korea; Portland State University, Portland, Oregon, USA

**Keywords:** bacteriophages, *Klebsiella pneumoniae*, phage, *Autographiviridae*, *Przondovirus*

## Abstract

Two *Klebsiella pneumoniae* bacteriophages, YMR1 and YMR2, which form plaques with halos, were isolated from sewage in Seoul, South Korea. YMR1 and YMR2 have double-stranded DNA genomes of 40,338 bp and 40,756 bp with 49 and 52 predicted protein-coding genes, respectively. Both are predicted to be members of the family *Autographiviridae*.

## ANNOUNCEMENT

*Klebsiella pneumoniae* is one of the ESKAPE pathogens with notorious virulence and antibiotic resistance (1, 2). Here, we investigated the genomes of two *K. pneumoniae*-infecting phages, YMR1 and YMR2. The phages were isolated from raw sewage samples (2 L; sedimented to remove sand) collected from the grit chamber of Tancheon and Joongnang Sewage Treatment Centers in Seoul, South Korea, on 8 and 9 February 2023, as described elsewhere with some modifications (3). Briefly, the sample was mixed with 2 × LB broth inoculated with host *K. pneumoniae* NCCP 16126 and NCCP 15864 and incubated at 37°C. After centrifugation (6,000 × *g*, 10 min) and filtration (0.45 µm pore-size filter), the supernatant was subjected to the plaque assay with the host *K. pneumoniae* culture to determine the presence of phages (4). A single plaque formed after overnight incubation was aseptically picked, eluted in SM buffer and repeated at least three times. The phages were propagated and concentrated by CsCl density-gradient ultracentrifugation as previously described (5).

Phage genomic material was extracted manually using the phenol-chloroform method (6). The Illumina library prepared for each phage using the TruSeq Nano DNA prep kit was sequenced using the Illumina Nextseq 2000 system (Illumina, San Diego, CA). Quality assessment was conducted using FastQC v0.11.9 (7). The adapter was trimmed using Trimmomatic v0.39 (8) and low-quality reads with Qscore <20 were removed. Contaminated reads were also filtered out using BWA v0.7.17 (9). The reads were then *de novo* assembled using SPAdes v3.15.2 (10), resulting in a single contig for each phage. Oligonucleotides targeting both ends of each contig were designed, followed by run-off Sanger sequencing to ensure a complete genome. Open reading frames (ORFs) were predicted using GenemarkS v4.28, GLIMMER-3, and FgenesB (11–13), and then annotated by searching for putative functions using BLAST and InterProScan v5.68-100.0 in parallel (14, 15). Functional assignments were categorized into five groups and visualized using GeneScene v0.99.8.0 (DNAstar, Madison, WI). tRNA genes were predicted using tRNA-ScanSE v2.0 (16), and genome termini were determined using PhageTerm v1.0.12 (17). Default parameters were used unless otherwise noted.

**Fig 1:**
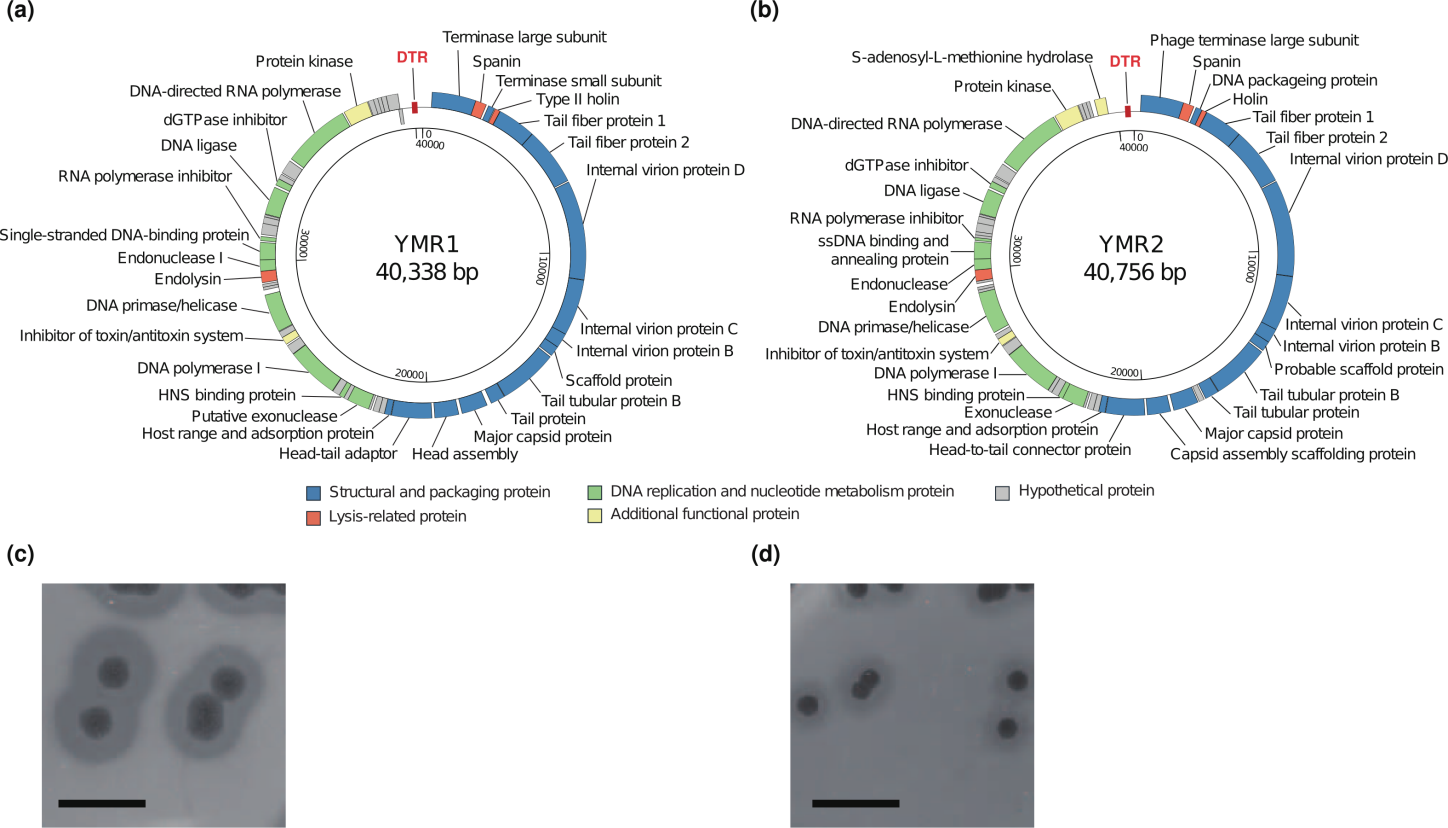
Genomic map and plaque morphology of phages YMR1 and YMR2. (**a and b**) The linear genome of each phage was plotted circularly. Colors are assigned to each putative protein-coding gene based on the expected functions of each gene product. DTR, direct terminal repeat region. Note that the start of the genome has been arbitrarily adjusted to facilitate comparison between two genomes. (**c and d**) Plaque morphology of phage YMR1 (**c**) and YMR2 (**d**) formed on the lawn of host *K. pneumoniae* NCCP 16126 and NCCP 15864, respectively. Note that each plaque was surrounded by halos. Scale bar, 10 mm.

The results of the complete genome sequencing for both phages are compared in Table 1. Two phages had similar genomic structures, but an ORF 49 encoding a hypothetical protein in YMR1 was transcribed in the opposite direction of the other genes (Fig. 1a and b). Both phages formed clear plaques with halos (Fig. 1c and d), suggesting possible depolymerase activity of their tail fiber proteins (18), but further studies are needed. The presence of DNA-directed RNA polymerase and comparative genomic analysis suggested their placement in the genus *Przondovirus*, family *Autographiviridae* (19). No genes for repressor, integrase, and antibiotic resistance in both phages, as well as clear plaque morphologies and lysis of *K. pneumoniae* culture, suggested their lytic nature and suitability as alternative anti-*K*. *pneumoniae* agents.

**TABLE 1:** Complete genome sequencing results for YMR1 and YMR2

	YMR1	YMR2
Raw reads	3,907,002	3,589,686
Read length (bp)	2 × 300	2 × 300
Total length of complete genome (bp)	40,338	40,756
GC content (%)	53.28	53.05
Fold coverage	21239.3×	21300.2×
Direct terminal repeats (bp)	179	181
No. of predicted protein-coding genes (functional; hypothetical)	49 (29; 20)	52 (19; 33)
No. of tRNA genes	0	0
Closest relative phage based on BLASTn analysis (GenBank accession no.; % similarity; % query coverage)	*Klebsiella* phage Pharr (NC_048175; 93.66, 92)	*Klebsiella* phage phiW14/TH1-302-1 (MT431699; 99.13, 95)

## Data Availability

The complete genome sequences of phage YMR1 and YMR2 have been deposited in GenBank under accession numbers PP662632 and PP662633, respectively. The associated BioSample and SRA accession numbers are SAMN41065711 and SRR28813546 for YMR1, and SAMN41107913 and SRR28847350 for YMR2, respectively. The associated BioProject accession number is PRJNA1103955.
